# Role of the Spleen Tyrosine Kinase Pathway in Driving Inflammation in IgA Nephropathy

**DOI:** 10.1016/j.semnephrol.2018.05.019

**Published:** 2018-09

**Authors:** Stephen McAdoo, Frederick W.K. Tam

**Affiliations:** Renal and Vascular Inflammation Section, Department of Medicine, Hammersmith Hospital Campus, Imperial College London, London, United Kingdom.

**Keywords:** Glomerulonephritis, cell signaling, tyrosine kinase, mesangial cell, B cell, Fc receptor

## Abstract

**Summary:** IgA nephropathy is the most common type of primary glomerulonephritis worldwide. At least 25% of patients may progress to kidney failure requiring dialysis or transplantation. Treatment of IgA nephropathy using generalized immunosuppression is controversial, with concerns regarding the balance of safety and efficacy in a nonspecific approach. This review describes the recent scientific evidence, and a current clinical trial, investigating whether spleen tyrosine kinase (SYK) may be a novel and selective therapeutic target for IgA nephropathy. SYK, a cytoplasmic tyrosine kinase, has a pivotal role as an early intermediate in intracellular signal transduction cascades for the B-cell receptor and the immunoglobulin Fc receptor, and thus is critical for B-cell proliferation, differentiation, and activation, and for mediating proinflammatory responses after Fc-receptor engagement in various cell types. In renal biopsy specimens of patients with IgA nephropathy, increased expression and phosphorylation of SYK were detected, and this correlated with the histologic features of mesangial and endocapillary proliferation. In cell culture studies, patient-derived IgA1 stimulated mesangial cell SYK activation, cell proliferation, and cytokine production, and these responses were attenuated by pharmacologic or molecular inhibition of SYK. A global, randomized, double-blind, placebo-controlled trial investigating the safety and efficacy of fostamatinib (an oral prodrug SYK inhibitor) in the treatment of patients with IgA nephropathy is ongoing, which may provide important evidence of the safety and efficacy of targeting this pathway in clinical disease.

Spleen tyrosine kinase (SYK) is an immunoreceptor-associated protein tyrosine kinase with numerous biological functions. First identified in 1991 (in lysates of porcine spleen, from whence it derives its name^1^), it now is known to be expressed in a variety of cell types, including fibroblasts, epithelial cells, and vascular endothelial cells,^2^ although at highest levels in hematopoietic cells, specifically B lymphocytes and myeloid cells. It has a well-characterized role in signaling for classic immunoreceptors, including the B-cell receptor (BCR) and activatory Fc receptors (FcRs), with an increasingly described role in other signaling pathways. As such, it has emerged as a potential therapeutic target in autoimmune and inflammatory disease. Here, we review the key functions of SYK and the evidence of its contribution to the pathogenesis of IgA nephropathy.

## STRUCTURE AND IMMUNE FUNCTIONS

**Figure 1:**
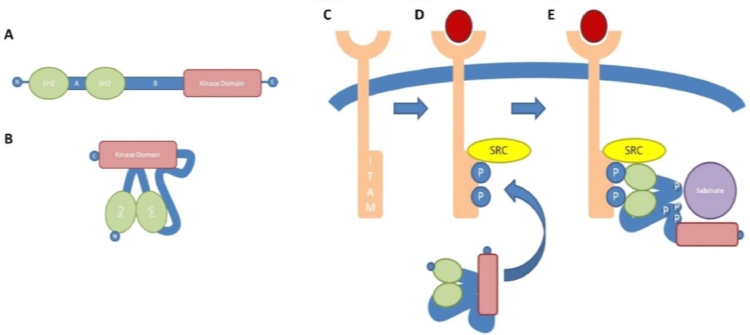
SYK structure and activation. (A) Schematic diagram showing the multidomain structure of SYK. (B) Schematic diagram of the linker-kinase sandwich conformation that has been suggested for inactive resting SYK. (C-E) SYK activation after interaction with ITAM. (C) Unengaged receptor bearing nonphosphorylated ITAM motif within the cytoplasmic domain. (D) Upon receptor engagement by corresponding ligand, Src family kinases (SRC) phosphorylate (P) tyrosine residues within the ITAM motif. (E) Phosphorylated ITAM acts as a docking site for the SH2 domains of SYK, resulting in conformational change, autophosphorylation and transphosphorylation of SYK tyrosine residues, and subsequent activation of downstream targets. Reprinted with permission from McAdoo and Tam.^65^

The SYK molecule has a multidomain structure containing two N-terminal tandem Src homology 2 (SH2) domains and a C-terminal kinase domain (Fig. 1A).^3^ The SH2-SH2 and SH2-kinase domains are linked by interdomains A and B, respectively. The molecule contains at least 10 major phosphorylation sites: 1 located in interdomain A, 5 within interdomain B, 2 within the kinase domain, and 2 near the extreme C-terminus.^4^ SYKB is an alternatively spliced form that lacks a 23 amino acid sequence in interdomain B, and in this respect is similar to ζ-chain–associated protein kinase 70 (ZAP-70), the only other member of the SYK family of kinases. ZAP-70 has approximately 60% overall homology to SYK and its expression appears to be more restricted, in particular to T lymphocytes and natural killer cells.^5^

In the resting state, it is thought that SYK assumes a closed, auto-inhibited structure, wherein interdomain A and interdomain B bind to the C-terminal kinase domain, preventing its interaction with potential substrates, in what has been termed a *linker-kinase sandwich* (Fig. 1B).^6^, ^7^ Upon activation, structural changes within the molecule result in an open conformation that allows the exposed catalytic kinase domain to interact with downstream targets. The canonical mechanism of SYK activation is via its interaction with immunoreceptor tyrosine-based activation motifs (ITAMs) (Fig. 1C).^8^ These are short consensus peptide sequences that contain two tyrosine residues 6 to 12 amino acids apart. As their name suggests, they are found in association with the cytoplasmic components of classic immunoreceptors, including the T-cell receptor, BCR, and FcR for immunoglobulins, either as an associated adaptor protein, or within the cytoplasmic region of the receptor itself.

Upon receptor engagement, the tyrosine residues on ITAMs are rapidly phosphorylated, primarily by Lyn and other members of the Src family of kinases that co-localize at the cell membrane (Fig. 1D). The phosphorylated ITAM can now act as a docking site for the SH2 domains of SYK, resulting in conformational changes, exposure of the kinase domain, autophosphorylation and transphosphorylation, and propagation of downstream signaling (Fig. 1E).

In addition to releasing the enzymatic domain of the protein from the linker-kinase sandwich, these changes in structure and phosphorylation, particularly within the tyrosine-rich interdomain B, create docking sites for downstream targets of SYK, for which it can perform both enzymatic and adaptor functions.^7^ These downstream targets include a host of adaptor proteins and other enzyme targets (including LAT, SLP76, Vav1, PLC-γ, phosphatidylinositol-3 kinase, and other mitogen-activated protein kinases) that are able to effect complex cellular responses including proliferation, differentiation, phagocytosis, and cytokine production.^9^

### Effects of SYK Knockout in vivo

The effects of germ-line SYK deletion in mice were reported in the mid-1990s.^10^, ^11^ SYK knockout resulted in perinatal death with a severe hemorrhagic phenotype. This subsequently was shown to be owing to a failure of communication between developing vasculature and lymphatics during embryogenesis.^12^ Analysis of bone marrow chimera animals, reconstituted with hematopoietic stem cells from SYK-deficient mice, showed impaired differentiation of the B-cell lineage (although relatively normal reconstitution of erythrocytes, platelets, myeloid cells, and T lymphocytes) with development arrested at the pro–B-cell to pre–B-cell stage, consistent with a role for SYK in pre-BCR signaling.^10^, ^11^ Subsequent in vitro work, using a variety of cell lines and cell-based reconstitution systems, defined a clear role for SYK in initiating downstream signaling after engagement of the BCR.^13^ More recently, conditional SYK deletion has allowed study of SYK function in mature B cells in vivo, and showed that B-cell maturation, long-term follicular B-cell survival, and antibody production are highly SYK dependent via both BCR and B cell activating factor-receptor–dependent mechanisms.^14^, ^15^

Analysis of myeloid cells from SYK knockout bone marrow chimeras showed ablation of FcR-mediated responses including phagocytosis and the generation of reactive oxygen intermediates.^16^, ^17^ The role of SYK in signal transduction for activatory FcR in these and a variety of other cell types is now well established, including mast cells bearing FcRε.^18^ Conditional SYK deletion confirms attenuation of FcR-mediated responses in myeloid cells, but not of FcR-independent processes, such as chemotaxis.^19^, ^20^ A critical role for SYK in FcRγ-mediated antigen internalization and maturation by dendritic cells also has been described, and is notable given the important role of dendritic cells in initiating adaptive immune responses.^21^

More recent attention has focused on the role of SYK in signaling for nonclassic immunoreceptors. SYK has been implicated in integrin signaling in myeloid cells, which is thought to be dependent on the association of integrins with ITAM-containing adapter proteins such as FcRγ chain and DAP12.^22^, ^23^ SYK also has a role in signaling for some innate pathogen recognition receptors, including those C-type lectin receptors that associate with ITAM-containing adapter proteins, and as such may have a role in antifungal immunity.^24^ Finally, SYK is implicated in a number of platelet activation pathways, including via the glycoprotein GPVI receptor (an FcRγ chain–associated receptor that bears an ITAM motif), integrin αIIbβ3, and C-type lectin 2 (a type II membrane protein containing a single tyrosine-based motif on its cytoplasmic tail that has been termed a *hemITAM*).^25^, ^26^

Given its important role in immunoreceptor signaling and in mediating immune and inflammatory responses, SYK function has been studied in a number of in vivo models of allergic and autoimmune disease. Studies using bone marrow chimeras generated using SYK-deficient progenitor cells, for example, confirmed that SYK deficiency in the hematopoietic compartment conferred resistance to a passively transferred antibody-dependent model of inflammatory arthritis.^27^ In addition, targeted genetic techniques, such as antisense oligonucleotides and small interfering RNA (siRNA) targeting SYK, have shown that it contributes to the pathogenesis of allergic airway inflammation and antibody-induced arthritis,^28^, ^29^, ^30^ and inducible SYK deletion using a Cre-loxP system confers protection to mast cell and myeloid cell–dependent models of inflammation.^19^, ^20^

### Pharmacological SYK Inhibition

A number of pharmaceutical companies are working to develop compounds to inhibit SYK for use in allergic and autoimmune disease.^31^, ^32^ A small number of these compounds have progressed to clinical studies, although to date published results in immune-mediated disease are available for only two such inhibitors, both developed by Rigel Pharmaceuticals (South San Francisco, CA): initially R112, and the related and more extensively studied compound, R406 (and its respective prodrug, R788; fostamatinib).^33^, ^34^ R406 is a competitive adenosine triphosphate inhibitor that binds to the catalytic domain of SYK, which has shown efficacy in animal models of immune thrombocytopenia,^35^ antibody-mediated arthritis,^33^, ^36^ and systemic lupus erythematosus,^34^, ^37^ with subsequent progression to clinical studies in immune thrombocytopenic purpura and rheumatoid arthritis.^35^, ^38^^,^^39^ Early phase studies using other novel SYK inhibitors, such as entospletinib and TAK-659, in the treatment of hematologic malignancy have been conducted,^40^, ^41^ although to our knowledge these agents have not been evaluated in the treatment of autoimmune conditions.

## SYK IN PATHOGENESIS OF IgA NEPHROPATHY

Current understanding of IgA nephropathy proposes a multihit model of disease pathogenesis.^42^ In genetically susceptible individuals, an inciting event, possibly mucosal infection, results in the production of galactose-deficient IgA1. An autoimmune response directed to this abnormal IgA1 molecule then is established, with the resultant formation of circulating immune complexes that may deposit in the renal mesangium, initiating a local inflammatory response that leads to glomerular damage and eventual glomerulosclerosis. SYK therefore may have a role in disease pathogenesis via its activity in IgA- and IgG-producing B cells or plasma cells, and/or in mediating the effects of the IgA1- and IgG-containing immune complexes when deposited in tissue.

### SYK Expression in Renal Biopsy Specimens in IgA Nephropathy

**Figure 2:**
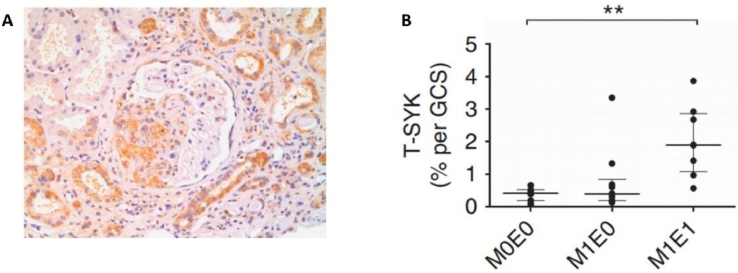
SYK expression in IgA nephropathy. (A) Immunoperoxidase staining for total SYK, showing SYK expression within an area of endocapillary glomerular proliferation and in distal tubular epithelial cells in a patient with IgA nephropathy. (B) Correlation of T-SYK detection with findings of mesangial proliferation (M1) and endocapillary proliferation (E1) in a cohort of patients with IgA nephropathy. ^⁎^*P* < 0.01. GCS,glomerular cross section. Reprinted with permission from McAdoo et al.^43^

Evidence for the latter phenomenon initially was provided by immunohistochemical analysis of renal biopsy specimens from patients with IgA nephropathy.^43^, ^44^ This was achieved by the use of antibodies detecting both splice variants of SYK (total SYK, T-SYK) and phosphorylated (activated)-SYK (P-SYK). T-SYK was detected in the glomeruli and tubules of patients with IgA nephropathy (Fig. 2A). In control patients with other causes of proteinuria (minimal change glomerulopathy) and hematuria (thin basement membrane glomerulopathy), expression of T-SYK was detected only in renal tubules. P-SYK was detected in the glomeruli but not in the tubules of patients with IgA nephropathy. Further studies showed that expression of T-SYK correlated with the severity of active IgA nephropathy (as defined by the presence of mesangial and/or glomerular endocapillary proliferation according to the Oxford Classification for IgA nephropathy) (Fig. 2B). The increased expression of SYK in IgA nephropathy was validated by Ryan et al^45^ in an independent study, in which they additionally observed that the number of glomerular P-SYK+ cells correlated positively with proteinuria and negatively with renal function in patients with IgA nephropathy.

### SYK Activation in Human Renal Mesangial Cells

**Figure 3:**
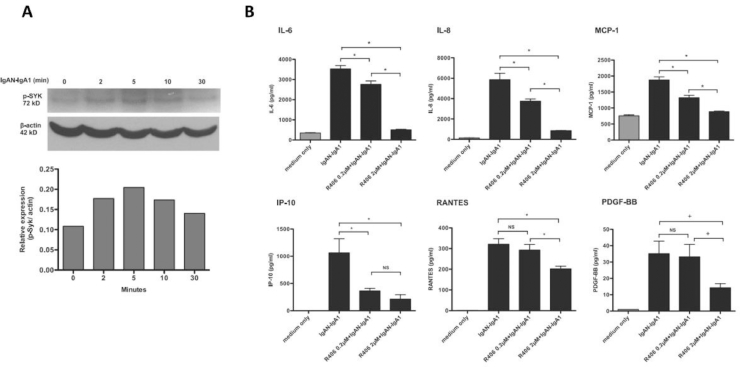
SYK activation and cytokine production by human mesangial cells after stimulation with patient-derived IgA1. (A) Time course of SYK activation (phosphorylation) in mesangial cells after stimulation by patient-derived IgA1. (B) Cytokine production by mesangial cells after stimulation by patient-derived IgA1, and dose-dependent reduction in cytokine production after incubation with selective SYK inhibitor R406, the active metabolite of fostamatinib. ^⁎^*P* < 0.05. IP, interferon gamma-induced protein 10. Reprinted with permission from Kim et al.^44^ Copyright 2012, the American Association of Immunologists, Inc.

The functional importance of SYK in IgA nephropathy initially was investigated using a cell culture model, wherein human glomerular mesangial cells were stimulated with IgA1 purified from patients with IgA nephropathy.^44^ Patient-derived IgA induced expression and phosphorylation of SYK (Fig. 3A), production of chemokines and proinflammatory cytokines (such as monocyte chemoattractant-1 [MCP-1], RANTES, interferon gamma-induced protein 10, interleukin [IL]-8, IL-6, and platelet-derived growth factor) (Fig. 3B), and cell proliferation. Purified IgA from healthy volunteers did not have this effect. These inflammatory and cell-proliferative effects of patient-derived IgA were inhibited by R406 (Fig. 3B). The specific role of SYK in these responses was confirmed further by siRNA knockdown in vitro.

The identity of the specific mesangial cell receptor(s) for IgA1 in patients with IgA nephropathy has been controversial, and a number of putative candidates have been suggested. The transferrin receptor (CD71) perhaps is the best characterized of these, being expressed on human mesangial cells, and up-regulated and co-localized with IgA1 deposits in IgA nephropathy.^46^, ^47^ Cross-linkage of CD71 by IgA can induce mesangial cell proliferation and cytokine production.^48^ CD71 blockade, however, only partially inhibits the binding of IgA to mesangial cells, implicating other potential receptors. FcRα (CD89) is known as a potential receptor for IgA, but its expression on human renal mesangial cells remains controversial.^49^, ^50^ Transfection and expression of human CD89 together with the human FcRγ chain was studied in a mouse mesangial cell line in vitro. In these transfected cells, stimulation with aggregated IgA resulted in activation of SYK and production of MCP-1.^51^ More recent theories have proposed that circulating soluble CD89 may form complexes with IgA that, in conjunction with transglutaminase 2, eventually binds to transferrin receptor (CD71) expressed on the surface of mesangial cells,^52^ although the involvement of SYK activation in this pathway is not known. In a recent study, Molyneux et al^53^ identified β1,4-galactosyltransferase 1 as a novel receptor of IgA1. Stimulation of this novel receptor with patient-derived IgA1 resulted in SYK phosphorylation and production of cytokine IL-6. The effect of IgA1 stimulation was reduced partially by inhibition of β1,4-galactosyltransferase 1. Of note, blockade of CD71 also partially inhibited the effects of IgA1 stimulation, although without additive effect when dual blockage of β1,4-galactosyltransferase 1 was performed, suggesting that these receptors may share a common intracellular pathway that recruits SYK.

The specific role of SYK in myeloid cells expressing the classic FcαR1 (CD89) in the pathogenesis of IgA nephropathy is less well studied. Historical data have suggested that glomerular and tubulointerstitial mononuclear phagocytic cell infiltration is associated with a worse prognosis in IgA nephropathy^54^; that CD89 expression on circulating leukocytes is up-regulated in IgA nephropathy patients, independent of plasma IgA levels, and that CD89 expression in glomeruli also may be increased compared with normal controls and patients with other forms of proliferative glomerulonephritis.^55^, ^56^ CD89 associates with FcRγ chain, an ITAM-containing adaptor protein, and multimerization of the receptor has been shown to activate SYK.^57^ In a recent study, serum IgA complexed with bacteria by opsonization-stimulated CD89 and SYK-dependent production of tumor necrosis factor-α.^58^ Our immunostaining studies suggested co-localization of SYK and CD68+ve in diseased glomeruli in IgA nephropathy,^43^ although the functional relevance of these findings to clinical disease will need further investigation.

### SYK in In Vivo Models of Proliferative Glomerulonephritis

A number of in vivo models of human IgA nephropathy have been described, including spontaneous, inducible, and genetically modified systems.^59^ However, the majority of these, in small rodents, are limited in that the glycosylation structure in the hinge region of IgA1 is unique to human beings and some nonhuman primates. In addition, although the rodent models may variably reproduce individual features of human disease (such as proteinuria, hematuria, or histopathologic changes), few show evidence of significant functional change or progression to glomerulosclerosis and end-stage renal disease. The effect of SYK deletion or inhibition has not, to the best of our knowledge, been studied in these possible models of IgA nephropathy.

Nevertheless, important lessons about the potential role of antibody-mediated inflammation have been learned in models of anti–glomerular basement membrane (GBM) antibody-mediated glomerulonephritis in rodents, which are reliably characterized by significant glomerular inflammation and progression to renal failure. In the passive-transfer model of anti-GBM glomerulonephritis in Wistar Kyoto rats (nephrotoxic nephritis), treatment with fostamatinib (the oral prodrug of R406) was effective in reducing macrophage infiltration, fibrinoid necrosis, and crescent formation, with a reduction in proteinuria and serum creatinine.^60^ Delayed treatment was effective, even if started after the onset of proteinuria. These therapeutic effects were associated with a reduction in the renal production of inflammatory cytokines, including MCP-1 and IL-1β. Cell culture studies showed that cytokine production stimulated by aggregated IgG occurred in both intrinsic glomerular cells (mesangial cells) and macrophages, and that these responses were suppressed by SYK inhibition with R406.

**Figure 4:**
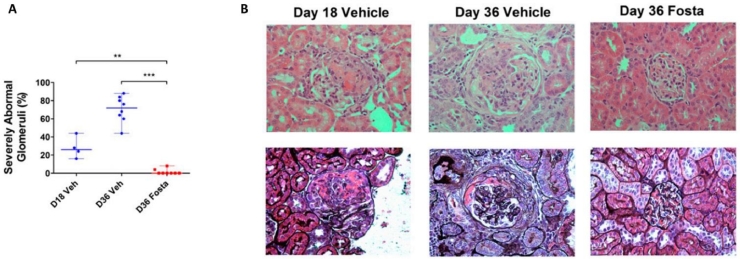
SYK inhibition in experimental autoimmune glomerulonephritis (EAG). (A) Reversal of glomerular injury after introduction of fostamatinib (Fosta) treatment at day 18 (D18) in EAG. By day 36 (D36) in vehicle-treated animals, there was progression of renal injury, whereas in fostamatinib-treated animals there was improvement in glomerular injury. (B) Representative histopathologic findings at day 18, and at day 36 after introduction of fostamatinib or vehicle treatment at day 18, showing established crescentic glomerulonephritis at day 18, with progression to circumferential fibrocellular crescent formation in vehicle-treated animals, and reversal to essentially normal glomerular findings in fostamatinib-treated animals. Upper panels show hematoxylin and eosin–stained sections; lower panels were stained with Jones methenamine silver stain to delineate glomerular and tubular basement membranes. ^⁎⁎^*P* < 0.01; ^⁎⁎⁎^*P* < 0.001. Reprinted with permission from McAdoo et al.^61^

In the active immunization model of anti-GBM glomerulonephritis (autoimmune experimental glomerulonephritis), disease is induced by immunization with the α3-chain of type IV collagen (the Goodpasture autoantigen), resulting in the development of autoantibody and crescentic glomerulonephritis. In this model, early treatment with fostamatinib reduced the production of autoantibody and the severity of glomerulonephritis.^61^ Late treatment with fostamatinib, commenced on day 18 when renal disease was well established, was effective in reducing microscopic hematuria, proteinuria, and histologic changes such as glomerular crescent formation. Comparison of histology with a control group on day 18 showed that the late treatment with fostamatinib was effective in reversing histopathologic injury in this model (Fig. 4). Ex vivo study of tissues from the rats with autoimmune experimental glomerulonephritis showed that pharmacologic inhibition of SYK reduced both production of autoantibodies by splenocytes, and the production of MCP-1, IL-12, and tumor necrosis factor-α within nephritic glomeruli. The importance of SYK in antibody-mediated glomerulonephritis in vivo was validated by the use of an alternative SYK inhibitor (synthesized by Celgene [Summit, NJ], and reported to inhibit SYK with partial activity against Flt3, KDR, and JAK2 in addition) in an acute model of nephrotoxic nephritis in rats.^62^ The relative contribution of myeloid-derived SYK was studied using selective deletion of myeloid cell SYK (predominantly in macrophages and neutrophils) in mice.^45^ Ryan et al^45^ found that myeloid cell knockout of SYK partially reduced the severity of nephrotoxic nephritis in mice. Taken together, these data suggest that treatment with a SYK inhibitor is effective in the reduction of antibody (when given early) and the reduction of the severity of glomerulonephritis (when given either early or late) in rodent models of antibody-mediated glomerulonephritis, and that these effects may be mediated via inhibition of SYK within both resident renal cells, such as mesangial cells, and within infiltrating leukocytes.

## OTHER CONSIDERATIONS

It is challenging to attribute the importance of a specific molecule or cytokine to the pathogenesis of glomerulonephritis, given that multiple complex, inter-related pathways may be involved. Taken together, however, the available preclinical data imply that SYK makes a significant contribution to the pathogenesis of IgA nephropathy, in particular via its role in mediating proinflammatory responses after the deposition of IgA1- and IgG-containing immune complexes in the renal mesangium. Emerging evidence suggests that the interaction of IgA1 with various receptors on mesangial cells results in SYK activation, although the potential of signaling via other receptors (including FcRγ, integrins, or C-type lectin receptors on resident on infiltrating cells) has yet to be explored specifically in IgA nephropathy. In addition, the role of SYK in the production of abnormal IgA1, and the autoantibodies directed against it, by B cells and plasma cells, has not been explicitly studied in IgA nephropathy, although data from other experimental immune models have suggested this may be a potential benefit, and other B-cell–directed therapies are undergoing clinical evaluation in IgA nephropathy. It is possible that a reduction in disease severity observed in the animal models of glomerulonephritis may be achieved by selective reduction (rather than complete abrogation) of SYK activity on these multiple pathways

Conversely, some of the therapeutic effects that have been observed when using small-molecule inhibitors may be attributed to off-target effects. R406 (the active metabolite of fostamatinib) is a potent inhibitor of SYK, although with a significant off-target effect on Flt3 and vascular endothelial growth factor receptor.^63^, ^64^ These off-target effects are less pronounced when studied in cell-based systems compared with isolated kinase assays, although the relative contribution of partial inhibition of multiple targets should be considered. It is encouraging, however, that the therapeutic effect seen in the experimental models has been shown by using two different SYK inhibitors by two different research groups, and that the in vitro effect in human mesangial cells has been validated by siRNA knockdown, and the in vivo effect by conditional SYK deletion.

## CLINICAL TRIAL OF FOSTAMATINIB IN IgA NEPHROPATHY: SYK INHIBITION FOR GLOMERULONEPHRITIS TRIAL

A proof-of-principle global clinical trial of fostamatinib was performed to study the efficacy and safety of fostamatinib in the treatment of patients with IgA nephropathy (NCT02112838). This was a randomized, double-blind, placebo-controlled, clinical trial that included a run-in period with optimized control of hypertension and proteinuria with angiotensin-converting enzyme inhibitor or angiotensin II receptor blocker for 90 days before randomization. The patients then were randomized to groups receiving fostamatinib 100 mg twice daily, 150 mg twice daily, or placebo for 24 weeks. This study completed global recruitment in February 2018, and results are expected later in 2018. Both functional (proteinuria, estimated glomerular filtration rate) and histopathologic end points will be evaluated because enrolled patients will be offered a repeat renal biopsy at the end of the treatment period. The results of this clinical trial will be pivotal in understanding whether SYK inhibition may be a safe and effective treatment of IgA nephropathy.
